# Peer Review of “Financial Feasibility of Developing Sustained-Release Incrementally Modified Drugs in Thailand’s Pharmaceutical Industry: Mixed Methods Study”

**DOI:** 10.2196/77627

**Published:** 2025-07-01

**Authors:** Elena Shkarupeta

**Affiliations:** 1 Voronezh State Technical University, Moskovskiy Prospekt, 14, Voronezh, Russian Federation, 7 473 255-37-76

**Keywords:** financial, economics, R&D, research and development, surveys, interviews, costs, revenue, policies, drugs, pharmaceuticals

*This is the peer-review report for “Financial Feasibility of Developing Sustained-Release Incrementally Modified Drugs in Thailand’s Pharmaceutical Industry: Mixed Methods Feasibility Study.”*

## Round 1 Review

### General Comments

This paper [1] presents a thorough analysis of the financial feasibility of developing incrementally modified drugs (IMDs) within the Thai pharmaceutical industry. It aligns well with Thailand’s National Strategic Master Plan and provides valuable insights for stakeholders regarding investment decisions and policy development. The mixed methods approach, including financial modeling, surveys, and interviews, lends credibility to the findings, while the focus on sustained-release dosage forms highlights a specific and practical application. The paper is well structured and contributes meaningfully to the discussion on enhancing local pharmaceutical capabilities. However, there are areas where clarity, presentation, and depth can be improved to strengthen its impact.

### Specific Comments

#### Major Comments

1. Clarity in objectives: While the paper provides an extensive background on Thailand’s pharmaceutical landscape, the research objectives could be more explicitly stated at the beginning of the introduction to guide the reader more effectively.

2. Discussion of results: The discussion section could delve deeper into comparing the financial feasibility of IMDs with other pharmaceutical products, especially generic drugs, to highlight the broader implications of the findings.

3. Policy recommendations: Although the paper suggests policy recommendations, it would benefit from providing concrete examples of how these policies have been successfully implemented in other regions or industries. This would add depth and context to the recommendations.

4. References and citation quality: The paper relies on only 15 references, which is insufficient for a study of this scope. Furthermore, only a few of these references are from peer-reviewed scientific journals, while the rest are reports and secondary sources. This significantly weakens the academic foundation of the study. It is strongly recommended to update the references section by incorporating recent, high-quality, and peer-reviewed articles.

#### Minor Comments

5. Terminology consistency: Terms like “incrementally modified drugs” and “IMDs” should be consistently used throughout the text to avoid confusion.

6. Figures and tables: Ensure all figures and tables are adequately labeled and referenced in the text. For instance, the presentation of financial data could be enhanced with clearer visualizations.

7. Formatting and grammar: Minor grammatical errors and formatting inconsistencies (eg, use of citations and spacing) should be addressed for a polished presentation.

8. Abstract refinement: The abstract could be more concise, emphasizing key findings and policy implications without overly detailed descriptions of methods.

9. Future research directions: Including a section on future research directions would enhance the paper’s utility for academics and policy makers.

## Abbreviations

IMD: incrementally modified drug
